# Trajectory of child food and nutrition policies

**DOI:** 10.1590/0102-311XEN094822

**Published:** 2023-08-28

**Authors:** Gilberto Kac, Maria Cláudia da Veiga Soares Carvalho, Nadya Helena Alves-Santos, Inês Rugani Ribeiro de Castro, Malaquias Batista

**Affiliations:** 1 Instituto de Nutrição Josué de Castro, Universidade Federal do Rio de Janeiro, Rio de Janeiro, Brasil.; 2 Instituto de Nutrição, Universidade do Estado do Rio de Janeiro, Rio de Janeiro, Brasil.; 3 Instituto de Estudos em Saúde e Biológicas, Universidade Federal do Sul e Sudeste do Pará, Marabá, Brasil.; 4 Instituto de Medicina Integral Professor Fernando Figueira, Recife, Brasil.

This interview composes the thematic issue on the nutritional transition, based on the *Brazilian National Survey on Child Nutrition* (ENANI-2019), but it is above all a tribute to Professor Malaquias Batista Filho, a reference in the fight against the ravages of hunger and child malnutrition in Brazil. His 87-year trajectory represents a milestone in public policies for food and nutrition in Brazil, not only for his resolve and principles, but also for the tenderness he has imprinted on this path. This interview seeks to record, based on his experience, the challenges faced these past decades to consolidate such policies by editing a videoconference with written answers sent by Professor Malaquias to previously sent questions.

Graduated in 1961, this physician from Paraíba is now a retired professor at the Federal University of Pernambuco (UFPE), but continues to work at the Institute of Integral Medicine of Pernambuco where, in 2020, he was awarded the title of “Cidadão Pernambucano” by the Legislative Assembly of the State of Pernambuco. He was a consultant to the Pan American Health Organization (PAHO), the United Nations Food and Agriculture Organization (FAO), and the United Nations Children’s Fund (UNICEF) for special missions in countries in Africa, Central America, and Latin America, including Brazil. Author (or co-author) of 74 books and 215 articles, he was awarded the Oswaldo Cruz Commendation by the Brazilian Ministry of Health, the National Food Safety Award by the Bunge Foundation, and the Anísio Teixeira Award by the Graduate Studies Coordinating Board (CAPES) of the Brazilian Ministry of Education in 2016.

Born in the rural area of São Sebastião do Umbuzeiro, in Cariri, Paraíba State, he graduated as a physician in 1961 from the Federal University of Paraíba, but had to move to Recife in 1966, fleeing political persecution by the military dictatorship. Teaching for 38 years at UFPE, he paved the way for the elaboration of public policies for food and child nutrition.

Right at the beginning, always very friendly and caring, Malaquias warned the interviewer - the Coordinator of ENANI-2019, Gilberto Kac - that his vision and hearing problems would hinder the virtual conversation, but he turned to Débora, his assistant, so as not to miss the dialogue. He spoke of the great pleasure of talking to Gilberto, and also with Nadya, project leader of ENANI-2019.

Gilberto Tell us a little about your academic background, your trajectory within Public Health, the paths you took, and your motivations for working in nutrition.

Malaquias About my academic background, my trajectory within Public Health, advances that have given me a certain professional notoriety, and political positions that even cost me police/military inquiries - all the relevant events are reported in a book that recovers the history of the Department of Nutrition at UFPE: *Como Nasceram Meus Anjos Brancos: A Constituição do Campo da Nutrição em Saúde Pública em Pernambuco* ^1^ [How My White Angels were Born: The Constitution of the Field of Nutrition in Public Health in Pernambuco], by Professor Francisco de Assis Guedes de Vasconcelos, from the Federal University of Santa Catarina. Fleeing the punitive action of the 1964 military dictatorship, which lasted 20 years, I had to sleep in the bush, in hammocks set up on the branches of *umbuzeiro* trees, or take shelter in the homes of relatives or acquaintances in Pernambuco, Recife State, and, in my state, Paraíba. This hidden curriculum, which also makes up my background, is recovered in other memorialistic documents, but should not be discussed at length here.

About Nutrition, I believe the story could be told from field studies. First, I resort to Josué de Castro himself, who in 1932 conducted a dietary survey with seven hundred families and, using today’s language, it was a hit, right? [laughs].

For lack of a more adequate space, this survey was published in the Official Gazette of Pernambuco: the initial statement about the situation of these seven hundred families filled one page ^2^ ^,^ ^3^.

Second, the multi-faceted survey that included housing cost, food cost, which accounted for fifty percent of the population’s cost of living approximately... it added transportation cost, clothing cost, and eventually leisure cost. I mean - at that time, Josué de Castro already had an integral view of the problems, that hunger is not just hunger, right? Flesh and blood people, people with body and spirit, let’s put it this way, have several other demands that go beyond food, although this was what caught Josué de Castro’s attention the most. And his book ended fundamentally by disclosing that. *Geografia da Fome* ^4^ [Geography of Hunger] and later *Geopolítica da Fome* ^5^ [Geopolitics of Hunger] were published only in 1946, giving visibility and acclaim to this study, in Brazil and worldwide.

Gilberto How would you describe the evolution of the nutritional epidemiological profile of children in the last four decades?

Malaquias Before the *Brazilian National Household Expenditure Survey* (ENDEF) [carried out in 1974] ^6^, and excluding Josué de Castro’s classic book (Geography of Hunger) ^4^, there is no safe baseline to define a consistent time horizon to demarcate a valid temporal reference to base a historical narrative.

Following the ENDEF, food and nutrition issues began to be perceived in a somewhat different way. This was a wary time, exactly because Brazil was not redefining itself with the overthrow of João Goulart’s government and the rise of the military. In São Paulo, Don Evaristo Arns publishes his book *São Paulo: Crescimento e Pobreza* ^7^ [São Paulo: Growth and Poverty], whose study group I could not join because of my leftist background... The ENDEF data was under heavy political scrutiny, right? And thus we see how Josué de Castro, or how many national data were politically silenced by dictatorship intervention, right?

Gilberto Was there censorship in publicizing the results on hunger and malnutrition under the military government?

Malaquias There were many eyes on the measurement data... first malnutrition had not dropped as the government, perhaps somewhat innocently, thought it would, right? This shows that the problem of hunger in Brazil has always been a forbidden topic, even when backed by numbers. We could thus discuss the issue of the silence imposed on Josué de Castro by military prohibitions, but that is a different matter.

Gilberto How important are the national surveys and, among them, the ENANI-2019 in this scenario?

Malaquias The safe resumption of the social-economic development process, with mitigation of the glaring and growing inequalities, adopted as a free policy by a government openly committed to the monopoly capital and, therefore, exclusionary. It is a crucial challenge, for it means changing the doctrinal anchor to a collectivist platform by prioritizing, for example, food and nutrition security as a fundamental right, as made explicit in the very statute of the Brazilian constitution. More than that: it is a universal cause of humanity itself.

Gilberto What are the key nutritional problems faced by children under five in Brazil today?

Malaquias The old problem of protein-energy malnutrition and its close or remote conditioning factors: the poverty or even indigence of many families, the unfavorable sanitation conditions of households and of the urban and rural environment, poor coverage and resolution of basic health actions, the parents’ low schooling level, the precarious social support network, early weaning, and the qualitative and quantitative improprieties of substitute or complementary feeding, and so on. These are the intricate “ecosystems of poverty” from which have resulted the complex clusters of nutritional deficiencies associated with various comorbidities. Brazil was already on the verge of overcoming the cycle of endemic shortages when the country escaped from the hunger map. Now the tables are turning, with indications that we may return to the tragic hunger map, not only affecting children, but adolescents, adults, and the elderly. We shall see if institutional conventions can save Recife and the world from these perverse prospects that could victimize tens of millions of people like a pandemic.

Low family income, unsanitary housing conditions, limited access to drinking water and sanitary sewage, low level of formal education of parents (especially mothers), difficult access and low resoluteness of basic health actions, inefficient social support network, advertising of processed foods for the immediate interest of the market rather than food security (healthy foods with adequate nutritional value), the practice of premature weaning, basic vaccination restrictions, inadequate control of health and nutrition indicators. Associated with this is the great problem of obesity, and many people do not realize that the obese person is a carrier of nutritional deficiencies. In short, these are impacts of the lack of food and nutrition security, which is a basic attribute of citizenship.

Gilberto In your opinion, what are the main determinants that help to understand and explain the current nutritional epidemiological scenario for Brazilian children?

Malaquias The previous question singularly points out some of the factors. In a generic and structural way, the so-called historical process of poverty, inequality, and therefore the absurdly asymmetrical sharing of production assets and wealth. It is a historical anomaly and one that only the reordering of the tortuous lines and forces of economic and social development can correct. This is crucial for all times, places and people.

Gilberto What food and nutrition policies do you highlight as important for improving the current scenario?

Malaquias At the short and medium term, to identify the people, families and social and biological groups most vulnerable and favor them with emergency and effective measures while these adversities prevail, regardless of their name: Bolsa Família Cash Transfer Program, Brazil Aid, Fight Against Hunger - as long as it is not focused on the remote future, but the millennium goals and objectives that were already in Josué de Castro’s head at least 75 years ago.

Gilberto For the future, what do you highlight as the main challenge to be overcome?

Malaquias The great challenge: how to correct the millennial and seemingly eternal story of ambition. A utopia? How to convert it into a practice of faith?

Gilberto Professor, these words mean a lot to me and it was a pleasure to have had the opportunity to spend some time with you. I thank you for your trajectory and all that you have represented to all of us, and still do. And I hope we can meet in person now that this pandemic is getting better. I also thank Deborah for being there beside you, facilitating our conversation. A big hug and thank you very much.

Malaquias Thank you very much, too. You represent the leadership that is in charge of renewing with enthusiasm, with intensity, with penetration, with impact the work of Josué de Castro... These are not empty words, but a fact. Keep up the good work!
